# Prognostic implications of immune-related eight-gene signature in pediatric brain tumors

**DOI:** 10.1590/1414-431X2020e10612

**Published:** 2021-05-17

**Authors:** Yi Wang, Chuan Zhou, Huan Luo, Jing Cao, Chao Ma, Lulu Cheng, Yang Yang

**Affiliations:** 1 Department of Neonatology and Neonatal Intensive Care, Zhumadian Central Hospital, Zhumadian, China; 2 Neonatal Intensive Care Unit, The Second Affiliated Hospital of Zhengzhou University, Zhengzhou, China; 3 Charité - Universitätsmedizin Berlin, Corporate Member of Freie Universität Berlin, Humboldt-Universität zu Berlin, and the Berlin Institute of Health, Berlin, Germany; 4 Department of Anatomy, College of Basic Medicine, Zhengzhou University, Zhengzhou, China; 5 Digital Medical Laboratory, Zhumadian Central Hospital, Zhumadian, China

**Keywords:** Pediatric brain tumor, Gene signature, Overall survival, Prognosis, Biomarkers

## Abstract

Genomic studies have provided insights into molecular subgroups and oncogenic drivers of pediatric brain tumors (PBT) that may lead to novel therapeutic strategies. Participants of the cohort Pediatric Brain Tumor Atlas: CBTTC (CBTTC cohort), were randomly divided into training and validation cohorts. In the training cohort, Kaplan-Meier analysis and univariate Cox regression model were applied to preliminary screening of prognostic genes. The LASSO Cox regression model was implemented to build a multi-gene signature, which was then validated in the validation and CBTTC cohorts through Kaplan-Meier, Cox, and receiver operating characteristic curve (ROC) analyses. Also, gene set enrichment analysis (GSEA) and immune infiltrating analyses were conducted to understand function annotation and the role of the signature in the tumor microenvironment. An eight-gene signature was built, which was examined by Kaplan-Meier analysis, revealing that a significant overall survival difference was seen, either in the training or validation cohorts. The eight-gene signature was further proven to be independent of other clinic-pathologic parameters via the Cox regression analyses. Moreover, ROC analysis demonstrated that this signature owned a better predictive power of PBT prognosis. Furthermore, GSEA and immune infiltrating analyses showed that the signature had close interactions with immune-related pathways and was closely related to CD8 T cells and monocytes in the tumor environment. Identifying the eight-gene signature (*CBX7*, *JADE2*, *IGF2BP3*, *OR2W6P*, *PRAME*, *TICRR*, *KIF4A*, and *PIMREG*) could accurately identify patients' prognosis and the signature had close interactions with the immunodominant tumor environment, which may provide insight into personalized prognosis prediction and new therapies for PBT patients.

## Introduction

Brain tumors are the most common solid tumor in pediatrics, accounting for 23.7% of new cancer diagnoses in children (1), and the second most common pediatric malignancy after leukemia (2,3). The life-saving treatments these children receive may result in impaired brain structure and function, leading to long-term major cognitive deficits (1,4–8). Current treatment options include surgical resection, cranial radiation, and chemotherapy. Survivors treated with high-load cranial radiation likely experience cognitive dysfunction, including difficulties in controlled attention, such as response inhibition and slower information processing (1,4–8). The distribution, pathology, molecular characteristics, and treatment strategies for pediatric brain tumors (PBT) have essential differences compared to those of the adult population (9).

Although the cure rate of PBT has increased in the past two decades of the 20th century, which was primarily due to advances in imaging, neurosurgery, and radiation oncology technologies and the introduction of combined chemotherapy, unfortunately, the overall survival has remained static (10). The lack of advances in PBT treatment was hindered by our lack of knowledge about the molecular pathogenesis of brain tumors (10). Advanced genomic analysis of the entire spectrum of PBT heralds an era in which this defect can be overcome by new technologies that will help us understand the genome pattern of PBT (10). Genomic studies have provided insights into molecular subgroups and oncogenic PBT drivers that may lead to novel therapeutic strategies (11).

The Gabriella Miller Kids First Data Resource Center (Kids First DRC; <https://kidsfirstdrc.org>) is a new, collaborative, pediatric research effort to understand the genetic causes and links between childhood cancer and structural congenital disabilities. This is a brand-new public database that has only been launched in recent years. It is vital that this data is expressly set up for children's tumor research, and few researchers have begun to mine this database.

Here, we conducted a comprehensive mining of the Kids First database to determine the minimum number of potentially robust genes that can be used to predict PBT patients' prognosis. Importantly, we used the LASSO algorithm, which can effectively analyze high-dimensional sequencing data (12 [Bibr B13] [Bibr B14]). Furthermore, we evaluated the accuracy of this eight-gene signature and validated it in a validation cohort. Moreover, gene set enrichment analysis (GSEA) and immune infiltrating analyses were conducted to explore the role of the signature in the tumor microenvironment.

## Material and Methods

### Data mining from the Kids First program

The gene expression profiles of PBT from 973 patients and their clinical and survival data were downloaded from Kids First Xena Hub (<https://kidsfirst.xenahubs.net>) with the cohort name: Pediatric Brain Tumor Atlas: CBTTC (CBTTC cohort). The study was conducted following the Declaration of Helsinki, and the Ethical Committee of Zhumadian Central Hospital approved the study.

### Identification and validation of the prognostic gene signature

First, all patients in the CBTTC cohort were randomly divided into training cohort (486 cases, 49.9%) and validation cohort (487 cases, 50.1%). In the training cohort, Kaplan-Meier analysis was used to screen the prognostic genes with a cutoff of P value <10.0E-15 in the log-rank test. Furthermore, univariate Cox regression analysis was performed on the training cohort to find prognostic genes with P values <10.0E-15. The intersected genes identified in Kaplan-Meier and univariate Cox analyses were then entered into the LASSO Cox regression model analysis, which was implemented in the training cohort utilizing R software (<http://www.r-project.org>) and the “glmnet” package (<http://cran.r-project.org>). Ten-time cross-validations were applied to detect the best penalty parameter lambda (12–15). According to the best lambda value, a list of prognostic genes with coefficients was obtained from the gene expression and patients' survival data.

Moreover, each patient's risk score was calculated based on the expression level of each prognostic gene and its corresponding coefficient. Using the median risk score as the cutoff point, the patients in the training cohort were distributed into high-risk or low-risk groups. Kaplan-Meier analysis was applied to evaluate the survival difference between the two groups. Cox and ROC analyses were conducted to further assess the gene signature's prognostic value in the training cohort. Furthermore, the prognostic gene signature was validated in the validation cohort. The same formula was conducted to compute risk scores like those in the training cohort. Kaplan-Meier, Cox, and ROC analyses were carried out as described earlier.

### Gene set enrichment analysis

The Hallmark (v7.1) and C7 (v7.1) gene set collections were downloaded from the Molecular Signatures Database v7.1 download page (https://www.gsea-msigdb.org/gsea/downloads.jsp). GSEA was performed based on the downloaded gene set collections using GSEA software (v4.0.3, https://www.gsea-msigdb.org/). The entire CBTTC cohort was taken to GSEA to reveal the functions and pathways in the differentially expressed genes between high-risk and low-risk groups. Only gene sets with family-wise error rate (FWER) P values <0.05 were considered significant.

### Correlation of risk score with the proportion of tumor-infiltrating immune cells (TICs)

The CIBERSORT (16) and MCP-counter (17) methods were used to estimate all tumor samples' TIC abundance distribution in the CBTTC cohort. The correlation was examined by the Spearman method.

### Statistical analysis

All statistical calculations were performed in the R software. Kaplan-Meier analysis was conducted to determine the overall survival differences between groups. Univariate and multivariate Cox proportional hazard regression analyses were conducted to assess the association between risk score and overall survival. The ROC analysis was applied to examine the sensitivity and specificity of survival prediction using the gene signature risk score. An area under the ROC curve (AUC) served as a pointer of prognostic accuracy. The R package “pROC” was used for ROC analysis, and the “Delong” method was used to study the significant differences among ROC curves. In all analyses, a P value <0.05 was considered statistically significant.

## Results

### Clinical characteristics

**Figure 1:**
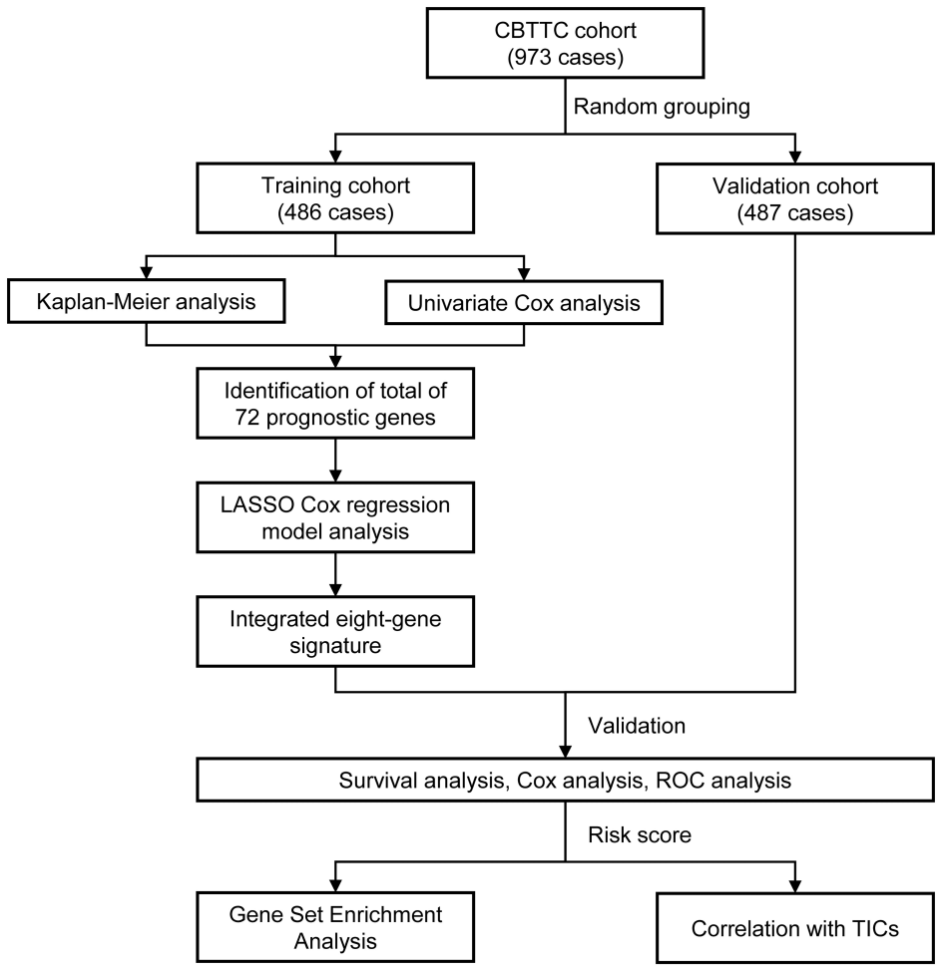
Flow chart of the study. The study was carried out in Pediatric Brain Tumor Atlas (CBTTC) cohort. The training cohort was used to identify prognostic genes. The LASSO regression model was used to establish a prognostic signature based on the prognostic genes. The prognosis analysis was validated in the validation cohort. LASSO: least absolute shrinkage and selection operator Cox regression model; ROC: receiver operating characteristic; TICs: tumor-infiltrating immune cells.

The flowchart of the present research is shown in Figure 1. A total of 973 cases in the CBTTC cohort were randomly distributed to a training cohort (N=486) and a validation cohort (N=487). The detailed clinical characteristics and grouping of these patients are summarized in Table 1.

**Table 1:** Clinical characteristics of 973 pediatric brain tumor patients involved in the study.

Characteristics	CBTTC cohort (N=973)	Training cohort (N=486)	Validation cohort (N=487)
Age at diagnosis, years			
<10	449 (46.14%)	209 (43.00%)	240 (49.28%)
≥10	381 (39.16%)	188 (38.69%)	193 (39.63%)
Unknown	143 (14.70%)	89 (18.31%)	54 (11.09%)
Sex			
Female	379 (38.95%)	189 (38.89%)	190 (39.01%)
Male	451 (46.35%)	208 (42.80%)	243 (49.90%)
Unknown	143 (14.70%)	89 (18.31%)	54 (11.09%)
Race			
American Indian or Alaskan Native	13 (1.34%)	5 (1.03%)	8 (1.64%)
Asian	28 (2.88%)	16 (3.29%)	12 (2.46%)
Black or African American	79 (8.12%)	32 (6.58%)	47 (9.65%)
More than one race	7 (0.71%)	3 (0.62%)	4 (0.82%)
Native Hawaiian or other Pacific Islander	2 (0.20%)	2 (0.41%)	0 (0%)
White	701 (72.05%)	339 (69.76%)	362 (74.34%)
Unknown	143 (14.70%)	89 (18.31%)	54 (11.09%)
Ethnicity			
Hispanic or Latino	28 (2.88%)	19 (3.91%)	9 (1.85%)
Not Hispanic or Latino	802 (82.42%)	378 (77.78%)	424 (87.06%)
Unknown	143 (14.70%)	89 (18.31%)	54 (11.09%)
Histological subtype			
Ependymoma	93 (9.56%)	44 (9.05%)	49 (10.06%)
Medulloblastoma	120 (12.33%)	57 (11.73%)	63 (12.94%)
Low-grade glioma/astrocytoma	252 (25.90%)	135 (27.78%)	117 (24.02%)
High-grade glioma/astrocytoma	109 (11.20%)	42 (8.64%)	67 (13.76%)
Others	399 (41.01%)	208 (42.80%)	191 (39.22%)

Data are reported as number and percent.

### Construction of prognostic signature from the training cohort

**Figure 2:**
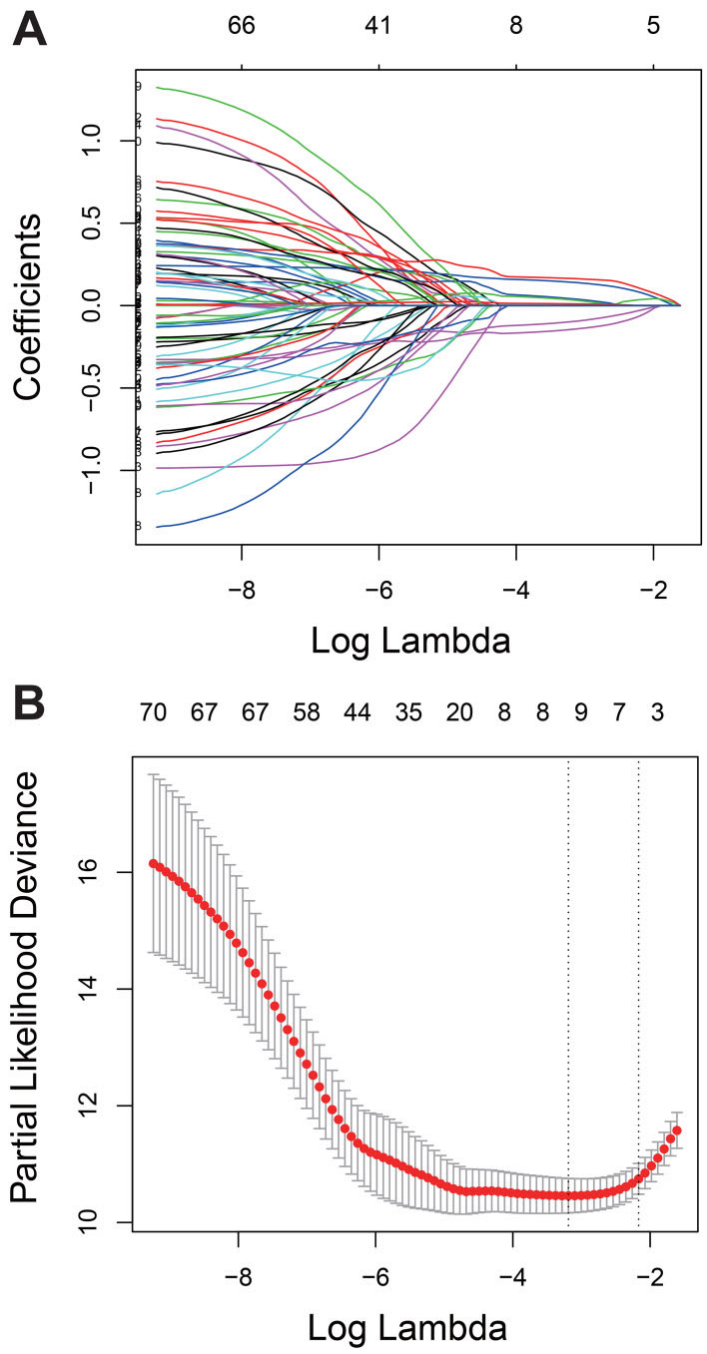
Establishment of prognostic gene signature by LASSO regression analysis. **A**, LASSO coefficient profiles of the 72 genes in the training cohort. **B**, Coefficient profile plot was generated against the log (lambda) sequence. Selection of the optimal parameter (lambda) in the LASSO model for training cohort. LASSO: least absolute shrinkage and selection operator Cox regression model.

Ninety-two genes were extracted from the Kaplan-Meier analysis (Table S1), while 136 genes were identified as significant in the Cox regression analysis (Table S2). Taken together, 72 genes in the intersection of the two results are defined as prognostic genes for subsequent analyses (Table S3). These prognostic genes were then subjected to LASSO Cox regression analysis, and regression coefficients were calculated. The coefficient of each gene is illustrated in Figure 2A. When 8 genes were included, the model achieved the best performance (Figure 2B). These genes and corresponding coefficients are shown in Table 2.

**Table 2:** Genes in the prognostic gene signatures.

Gene symbol	Full name	Risk coefficient
*CBX7*	Chromobox 7	-0.097248966
*IGF2BP3*	Insulin Like Growth Factor 2 MRNA Binding Protein 3	0.141763903
*JADE2*	Jade Family PHD Finger 2	-0.144454955
*KIF4A*	Kinesin Family Member 4A	0.041651464
*OR2W6P*	Olfactory Receptor Family 2 Subfamily W Member 6 Pseudogene	0.034386859
*PIMREG*	PICALM Interacting Mitotic Regulator	0.004196815
*PRAME*	Preferentially Expressed Antigen in Melanoma	0.010705036
*TICRR*	TOPBP1 Interacting Checkpoint and Replication Regulator	0.164511862

### Prognostic value of the eight-gene signature in the cohorts

**Figure 3:**
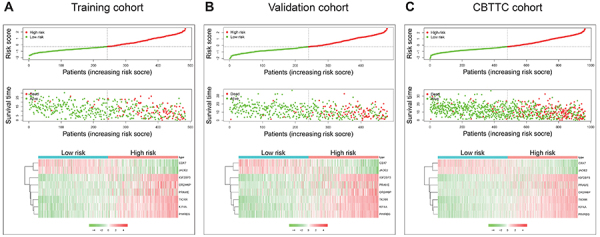
Characteristics of the eight-gene signature. Upper and middle, the distribution of risk score and patient's survival time for training (**A**), validation (**B**), and Pediatric Brain Tumor Atlas (CBTTC) (**C**) cohorts. The black dotted line is the median cutoff dividing patients into low-risk and high-risk groups. Bottom, heatmap of the eight-gene expression profiles for prognostic signature for training, validation, and CBTTC cohorts.

The distribution of risk scores and survival status and the expression profiles of the eight-gene signature of the patients in the training cohort were plotted and are shown in Figure 3A. As shown in the figure, there were more deaths in the high-risk patient group, and the survival time was shorter than that of the low-risk patient group. The heatmap indicates that *CBX7* and *JADE2* were under-expressed in high-risk patients, while *IGF2BP3, OR2W6P, PRAME, TICRR, KIF4A,* and *PIMREG* were highly expressed in high-risk patients. We also verified the performance of this eight-gene signature in the validation cohort. As shown in Figure 3B and C, the pattern was consistent with that in the training cohort. Furthermore, we examined this eight-gene signature in subtypes in the CBTTC cohort and the results were similar (Figure S1).

**Figure 4:**
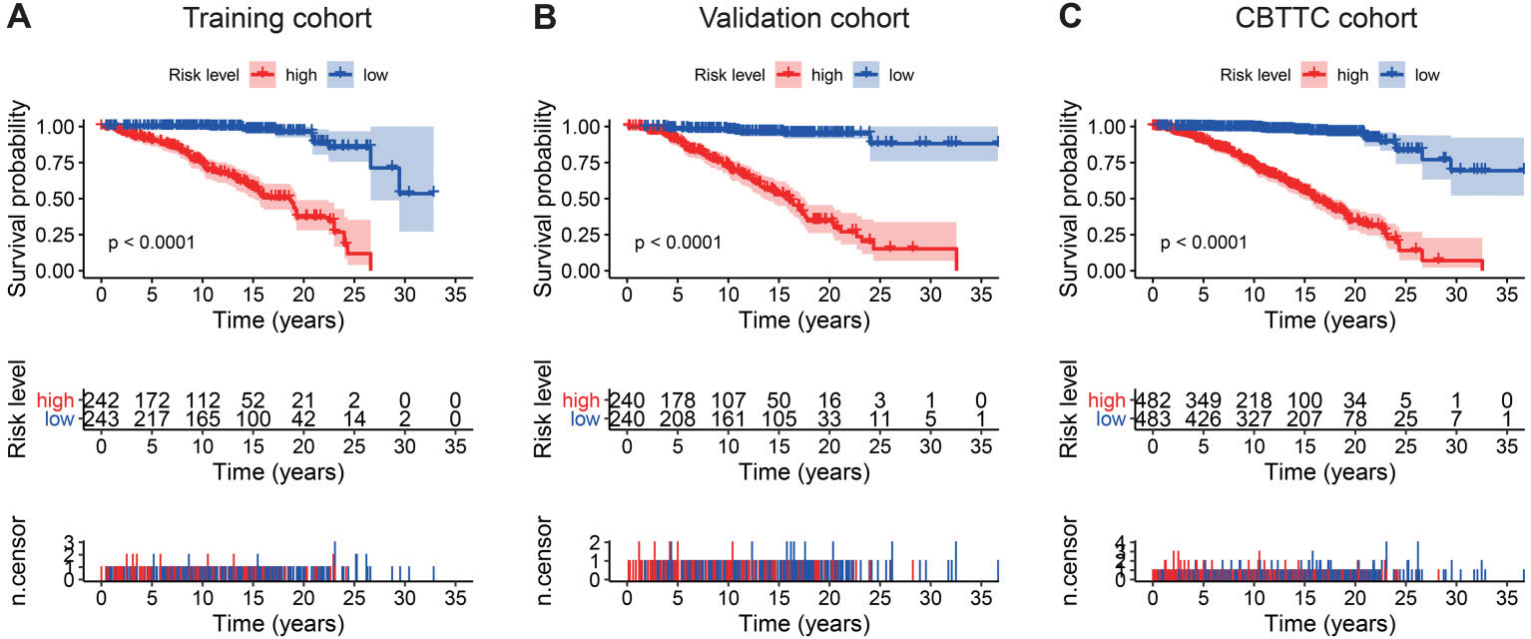
. Kaplan-Meier analyses of the overall survival based on the eight-gene signature. **A**, Training cohort. **B**, Validation cohort. **C**, Pediatric Brain Tumor Atlas (CBTTC) cohort. The differences between the two curves were determined by the two-side log-rank test. The number of patients at risk are listed in the middle plot of each cohort.

Kaplan-Meier survival analysis showed that patients in the high-risk group were associated with a poor prognosis trend in the training cohort (P value <0.0001, Figure 4A). To confirm the efficacy of the eight-gene signature in predicting overall survival in PBT patients, it was examined in the validation cohort. According to the median risk score, patients were divided into high-risk and low-risk groups using the same classification method. Consistent with previous results, patients in the high-risk group showed significantly worse overall survival than patients in the low-risk group (P value <0.0001, Figure 4B). In the entire CBTTC cohort, which was the sum of the training and validation cohorts, the eight-gene signature also had similar predictive ability (P value <0.0001, Figure 4C). We tested the capacity of each of the eight genes via Kaplan-Meier analysis and found CBX7 and JADE2 predicted favorable outcomes, while the remaining genes had poor effects on the prognosis (Figure S2). Moreover, the subtypes of PBT in the CBTTC cohort were also examined by Kaplan-Meier analysis, which showed that the eight-gene signature risk score predicted the survival of PBT subtypes (Figure S3).

**Figure 5:**
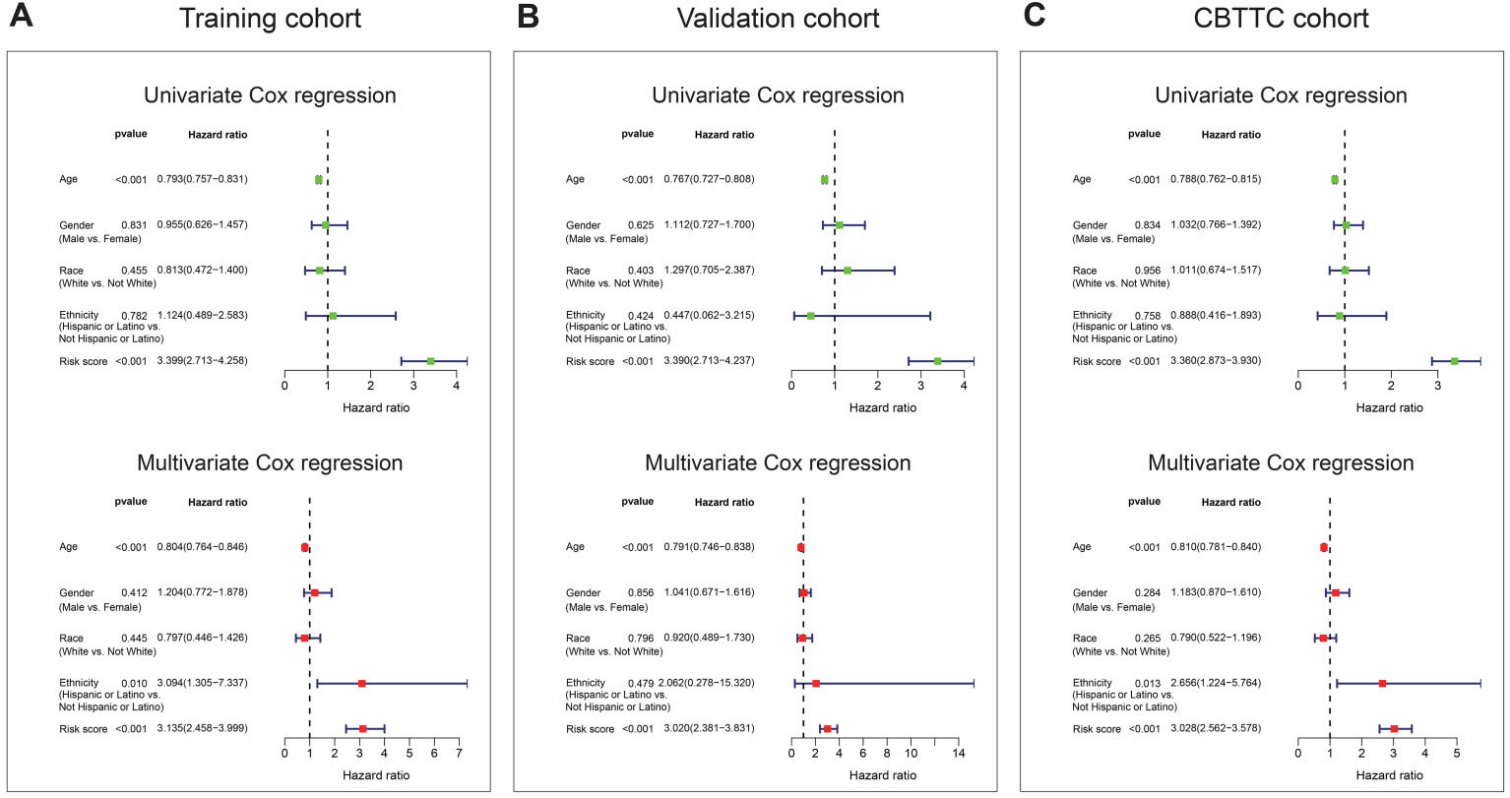
. Forest plot summary of overall survival analyses. Univariate (upper) and multivariate (bottom) analyses based on the eight-gene signature and clinical covariates in training (**A**), validation (**B**), and Pediatric Brain Tumor Atlas (CBTTC) (**C**) cohorts. The colored solid squares represent the hazard ratio (HR), and the transverse lines through HR represent 95% confidence intervals (CI). All P values were calculated using Cox hazards regression analysis.

Univariate and multivariate Cox analyses were performed in the training, validation, and CBTTC cohorts, using the available co-variables including risk score, age, gender, race, and ethnicity to confirm whether the prognostic capacity of our eight-gene signature was independent from the clinic-pathologic characteristics. In the training cohort, both univariate and multivariate Cox regression analyses indicated that the eight-gene signature was a powerful variable associated with prognosis (HR=3.399, 95%CI: 2.713-4.258, P value <0.001 and HR=3.135, 95%CI: 2.458-3.999, P value <0.001, respectively; Figure 5A). Consistent with that in the training cohort, the eight-gene signature displayed a similar ability in the validation and CBTTC cohorts (Figure 5B and C). These results proved that the eight-gene signature was a strong and independent variable.

**Figure 6:**
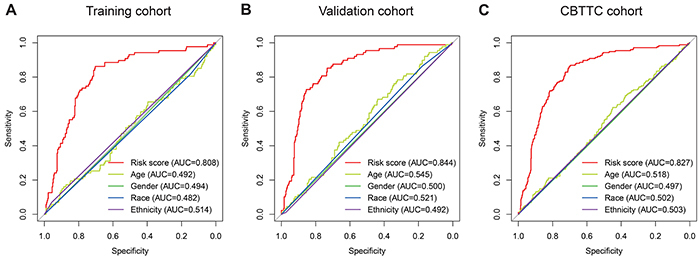
Receiver operating characteristic (ROC) analysis of the eight-gene signature risk score. ROC analysis of the sensitivity and specificity of the overall survival prediction by the eight-gene risk score, age, gender, race, and ethnicity in the training (**A**), validation (**B**), and Pediatric Brain Tumor Atlas (CBTTC) (**C**) cohorts. AUC: area under the ROC curve.

Subsequently, we conducted ROC analyses to assess how the eight-gene signature would behave in predicting prognosis. As shown in Figure 6A, the area under the ROC curve (AUC) of the eight-gene risk score model performed in the training cohort was 0.808, which was superior to those of age, gender, race, and ethnicity (0.492, 0.494, 0.482, and 0.514, respectively). This finding was also confirmed in the validation cohort (AUC=0.844, Figure 6B) and CBTTC cohort (AUC= 0.827, Figure 6C).

### Gene set enrichment analysis with the eight-gene signature

**Figure 7:**
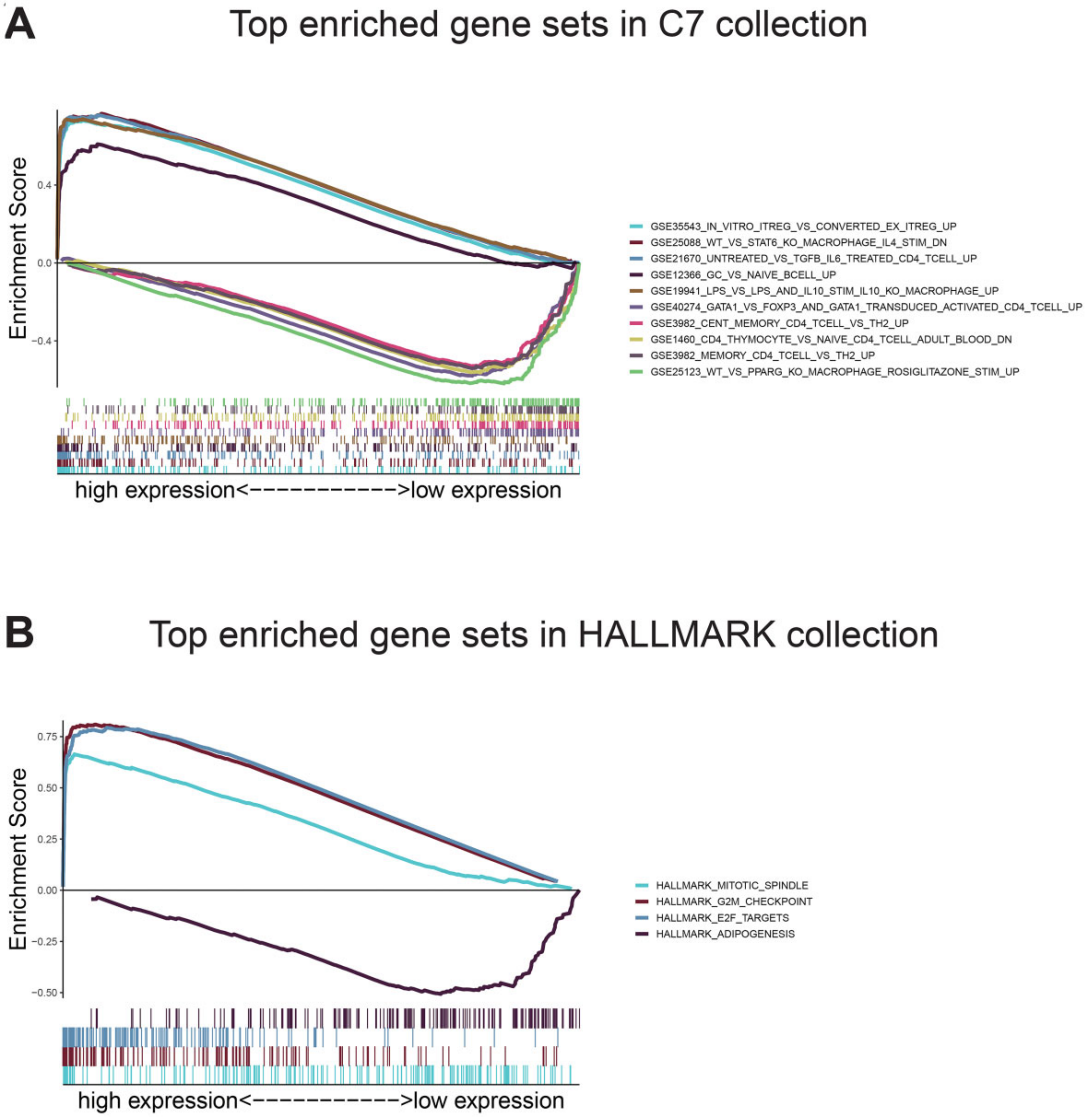
Gene Set Enrichment Analysis. **A**, Top ten enriched gene sets annotated by the C7 collection between the high- and low-risk groups in the CBTTC cohort. **B**, Enriched gene sets annotated by the HALLMARK collection between the high- and low-risk groups in the CBTTC cohort. Each line represents one specific gene set with unique color. Up-regulated genes are located at the left, approaching the origin of the coordinates, and the down-regulated are located at the right of the x-axis. Only gene sets with family-wise error rate (FWER) P values <0.05 were considered significant.

Given the negative correlation between the eight-gene signature risk score and the overall survival of PBT patients, GSEA was conducted between the high- and low-risk groups. As displayed in Figure 7A and Table S4, genes in the high-risk group were mostly enriched in immune-related functions and pathways. They were relating to regulatory T cells, macrophages, CD4 T cells, TGF beta, IL6, and naive B cells. As to the low-risk score group, the genes were enriched in pathways involved in macrophages, CD4 T cells, T helper type 2 cells, and FOXP3+ regulatory T cells, which were also closely immune-related (Figure 7A and Table S5). For HALLMARK collection defined by the Molecular Signatures Database, multiple immune functional gene sets like HALLMARK_MITOTIC_SPINDLE, HALLMARK_G2M_CHECKPOINT, and HALLMARK_E2F_TARGETS were significantly enriched in the high-risk group (Figure 7B and Table S6); whereas only one gene set HALLMARK_ADIPOGENESIS was enriched in the low-risk group (Figure 7B and Table S7). These findings indicated that the risk score was potentially closely related to the status of tumor microenvironment.

### Correlation of risk score with the proportion of TICs

**Figure 8:**
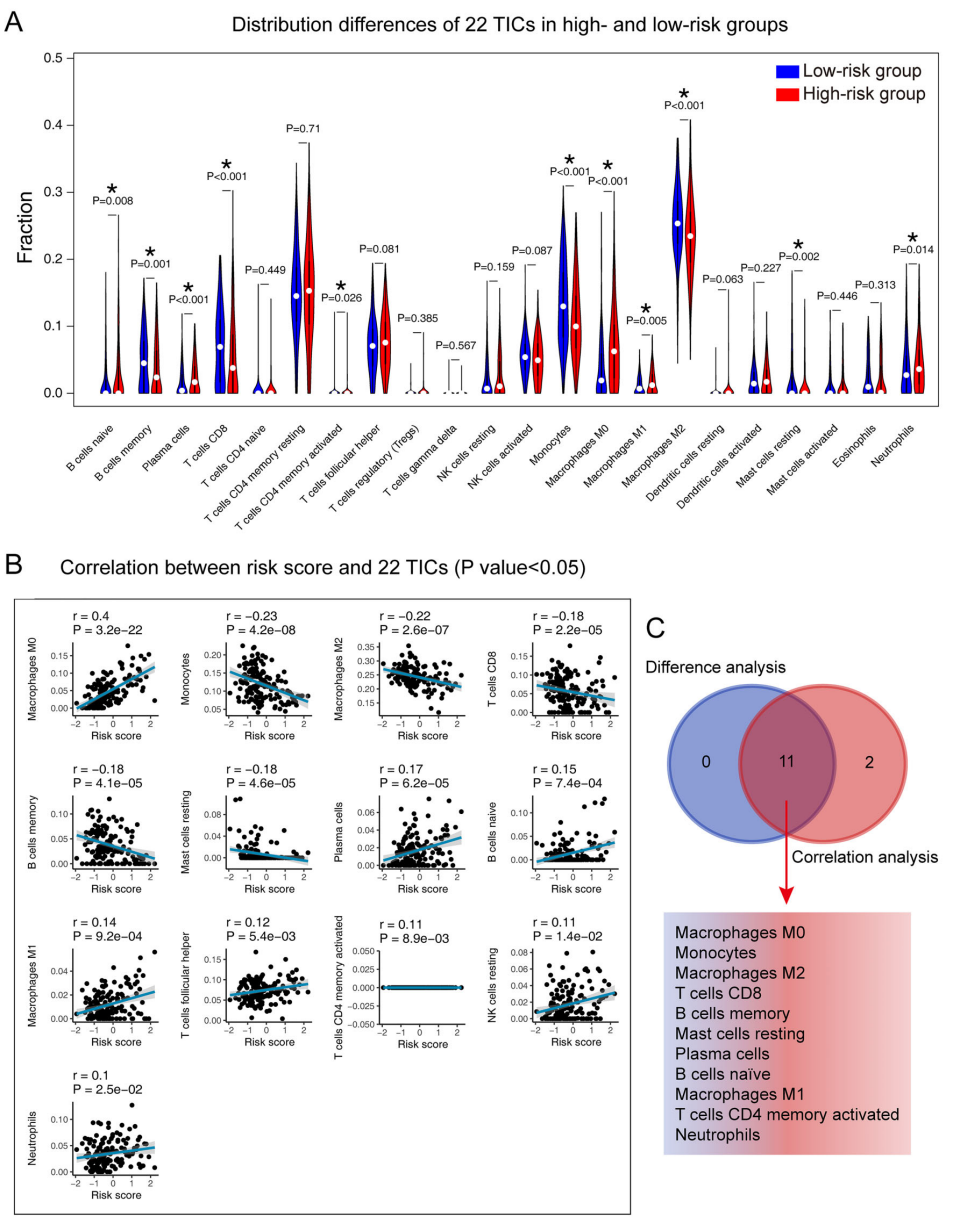
Correlation of TICs proportion with the eight-gene signature risk score in the Pediatric Brain Tumor Atlas (CBTTC) cohort (CIBERSORT method). **A**, Violin plot showing the ratio differentiation of 22 kinds of immune cells between PBT samples from low- and high-risk groups to the median risk score. Wilcoxon rank sum test was used to assess for significance. **B**, The blue line in each plot is the fitted linear model indicating the proportion tropism of the immune cell with risk score. The shade around the blue line represents the 95% confidence interval. Spearman coefficient was used for the correlation test. **C**, Venn plot displayed 11 TICs correlated with risk score co-determined by difference and correlation tests shown in violin and scatter plots, respectively. P value <0.05 was the cutoff. TIC: tumor-infiltrating immune cell; PBT: pediatric brain tumor.

**Figure 9:**
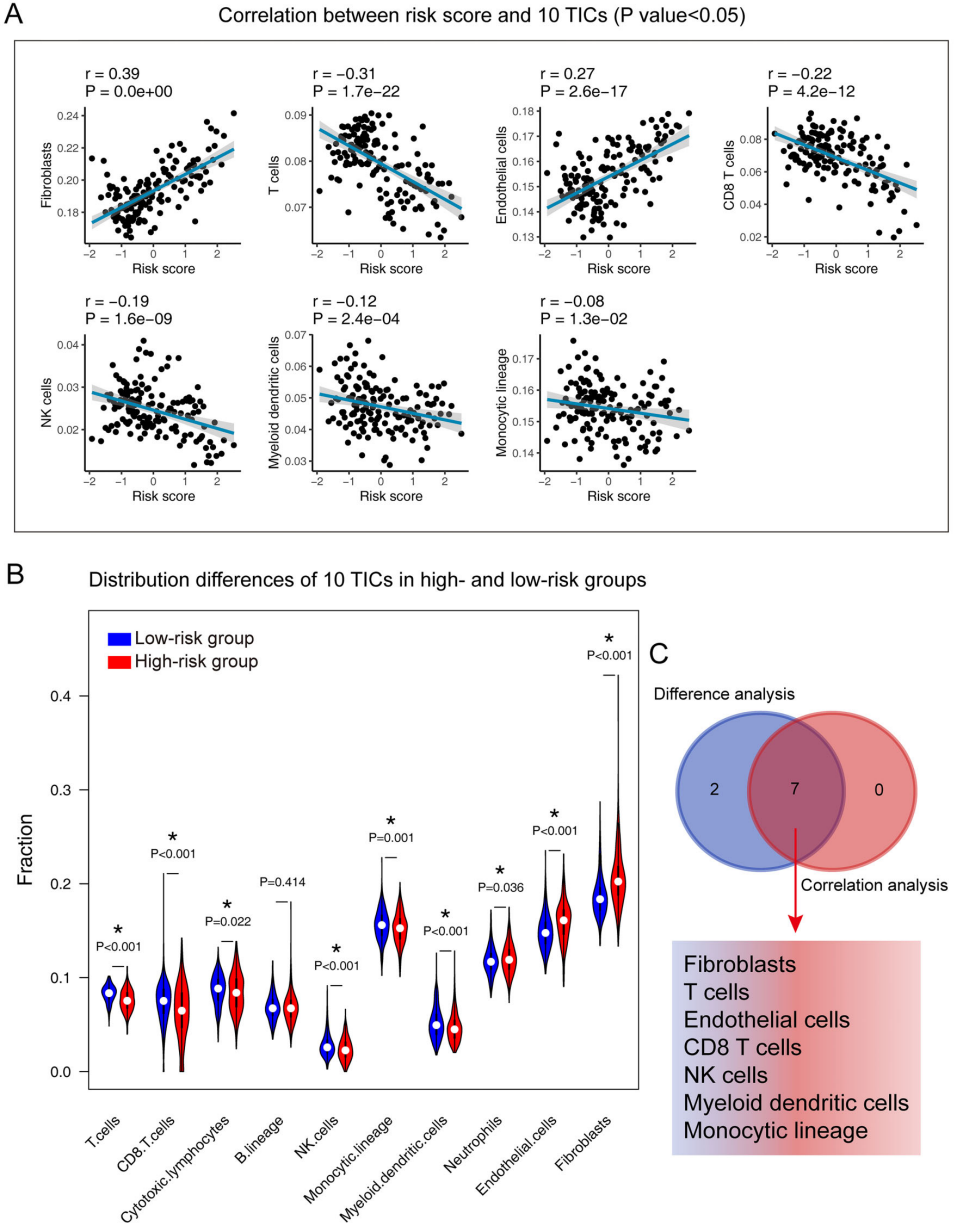
Correlation of TICs proportion with the eight-gene signature risk score in the Pediatric Brain Tumor Atlas (CBTTC) cohort (MCP-counter method). **A**, The blue line in each plot is the fitted linear model indicating the proportion tropism of the immune cell with risk score. The shade around the blue line represents the 95% confidence interval. Spearman coefficient was used for the correlation test. **B**, Violin plot showing the ratio differentiation of 10 types of immune cells between PBT samples from low- and high-risk groups to the median risk score. Wilcoxon rank sum test was used to assess for significance. **C**, Venn plot displays 7 TICs correlated with risk score co-determined by difference and correlation tests shown in violin and scatter plots, respectively. P value <0.05 was the cutoff. TIC: tumor-infiltrating immune cell; PBT: pediatric brain tumor.

To further confirm the correlation between the risk score and the immune microenvironment, as shown in Figure S4 and Figure S5, we used the CIBERSORT and MCP-counter algorithm to analyze the proportion of TICs subpopulations and constructed immune cell profiles in PBT samples. Combining the results of correlation analysis (Figure 8A) and differential analysis (Figure 8B), a total of 11 TICs were associated with the eight-gene signature risk score (Figure 8C). In the result of the MCP-counter algorithm (Figure 9), a list containing 7 TICs identified closely with the signature. In summary, by adopting these methods, CD8 T cells and monocytes were overlapped in the two results and seen as critical cells that affected the eight-gene signature in the tumor environment of PBT.

## Discussion

At present, diagnosis, prognosis, and treatment of PBT are highly dependent on the histopathological characteristics of the tumor (3,18). However, more importantly, given the current development of precision medicine and genetic research of tumors, in the past decade, significant changes have taken place in pediatric neuro-oncology, and exploring optimized tumor biomarkers will be the trend of future development (19,20). In this study, we built a PBT prognostic signature by comprehensively analyzing the Kids First database, designed to understand the genetic causes and connections of childhood cancer and congenital structural disabilities.

After we constructed the eight-gene signature, we firstly confirmed its capacity to distinguish the survival time and survival status of patients effectively. As shown in Figure 3A, the high-risk zone not only counted more deaths, but also the patients in it presented a shorter survival time than those in the low-risk zone. Moreover, the heatmap indicated that each of these eight genes had a differential expression pattern between the low- and high-risk groups. Importantly, this eight-gene signature had the same or similar performance in the validation cohort (Figure 3B) and the CBTTC cohort (Figure 3C).

In addition, we examined the prognostic value of the eight-gene signature by Kaplan-Meier analysis in training, validation, and CBTTC cohorts, finding its predicting ability in PBT patients significant (Figure 4). Furthermore, univariate and multivariate analyses were performed in the three cohorts to confirm whether our eight-gene signature could be independent from other variables in predicting PBT overall survival. As plotted in Figure 5, no matter in which cohort, whether it was univariate or multivariate Cox regression analysis, the variable of risk score was always statistically significant, and the results confirmed the predictive ability of the risk score, and its independence.

To further assess the predictive power of this eight-gene signature, ROC analysis was conducted. In diagnostic tests, AUC is used to check accuracy and determine the predictive capacity of biomarkers (21). ROC analysis indicated that the AUC of the eight-gene signature stayed above 0.8 in these three cohorts and was superior to age, gender, race, and ethnicity. These ROC results again suggested that our signature strengthened the predictive accuracy of prognosis in PBT.

Our signature was composed of eight genes, which are *CBX7, JADE2, IGF2BP3, OR2W6P, PRAME, TICRR, KIF4A,* and *PIMREG*. In the signature model, *CBX7* and *JADE2* were protective genes, whereas other genes were unfavorable on the overall survival of PBT patients. *IGF2BP3* is an oncofetal protein that binds RNA, thereby influencing the fate of target transcripts, and it is up-expressed in a variety of malignant tumors and represents a promising cancer biomarker (22). *PRAME* is a tumor-associated antigen that was first identified through analysis of the specificity of tumor-reactive T-cell clones derived from a patient with metastatic cutaneous melanoma (23). Subsequently, it was found that *PRAME* is not only expressed in cutaneous melanoma, but also in ocular melanoma and various non-melanoma cell malignancies (24). *PRAME* expression can be detected in 82% of medulloblastoma samples, regardless of molecular and histopathological subgroups. The high expression of *PRAME* is also related to poor outcomes of patients with medulloblastoma. Studies have shown that adoptive immunotherapy that redirects T cells to *PRAME* antigen may represent an innovative treatment for medulloblastoma (25). *TICRR* is one of the important replication initiation factors. The knockout of *TICRR* significantly inhibits tumor cell growth, migration, and colony formation *in vitro*, and inhibits tumor growth in xenograft models (26). A recent study demonstrated that *TICRR* is a major carcinogen, which can accelerate the proliferation of cancer cells by promoting the initiation and progression of DNA replication (27). *KIF4A* was found to be implicated in the regulation of chromosome condensation and segregation during mitotic cell division, which is essential for eukaryotic cell proliferation (28). *KIF4A* is aberrantly expressed in a variety of cancers, and it is overexpressed in most tumors but also low-expressed in a few tumors, suggesting distinct functions and mechanisms for different tumors (29,30). *PIMREG* plays a key role in regulating cell proliferation and is induced by mitogens, and its protein level is related to the cell cycle (31). Jiang et al. found that *PIMREG* played a crucial role in the promotion of breast cancer aggressiveness via constitutive activation of the NF-κB signaling pathway (32). There are relatively few studies related to these genes and PBT. However, the eight-gene signature had a significant role in predicting and diagnosing PBT in our research. The eight-gene signature or each of them may indicate specific directions for future research on PBT.

The findings of the GSEA analysis demonstrated that the eight-gene signature might potentially participate in the immune-dominant tumor microenvironment. The analysis based on CIBERSORT algorithm for the proportion of TICs showed that half of TICs (11/22) were correlated with the eight-gene signature risk score in PBT patients, further supporting that the signature interacted closely with the tumor environment. Combining the CIBERSORT and MCP-counter methods, we found CD8 T cells and monocytes were in a close relationship with the eight-gene signature in the tumor environment of PBT. Strategies targeting the tumor microenvironment of pediatric brain cancers have the potential to improve the efficacy of standard and genome-based molecular therapeutics and to help resolve many of the challenges associated with developing new drugs and running clinical trials for a relatively small PBT population (33). The specific pathways and TICs revealed in our analysis have potential for tumor microenvironment targets in further studies.

Our research also had some limitations. For the study of PBT, currently available public databases are very limited. The datasets in GEO and TCGA are not eligible for validation purposes because of the population's age distribution. Our eight-gene signature came from retrospective data, and more prospective data is needed for proving the clinical utility of it. In addition, due to the very limited clinical characteristics of patients included in the CBTTC cohort, we could not perform certain clinical subgroup analyses. Furthermore, there are currently no wet experimental data explaining the relationship between these eight genes and their mechanism in PBT samples. Therefore, more research is needed to clarify the potential relationship.

In conclusion, our research defined a robust eight-gene signature in PBT. It was a comprehensive analysis of the new Kids First database. This signature was related to PBT's overall survival and accurately identified the prognostic risk of patients. Notably, we assessed the reliability and accuracy of the signature in a validation cohort. In addition, the functions and immune infiltrating analyses showed that the signature had close interactions with CD8 T cells and monocytes in the tumor environment, which may advance the development of new therapies for PBT treatment.

## Supplementary Material

Click here to view [pdf].
